# Supplementary material for: Detection of Fused Genes in Eukaryotic Genomes using Gene deFuser: Analysis of the Tetrahymena thermophila genome
Source: BMC Bioinformatics. 2011 Jul 11;12:279. doi: 10.1186/1471-2105-12-279 (PMC3143110; doi:10.1186/1471-2105-12-279)
Supplement: Additional file 1 — Results of Gene deFuser for the Tetrahymena thermophila genome. This zip file contains the raw results of the analysis of the Tetrahymena genome using Gene deFuser. To view the contents, unzip the file and open the Final_Tet.html file in the resulting folder. [file 1471-2105-12-279-S1.ZIP › Results/3692.m00067.html]

Gene deFuser -- Results of Job Final\_Tet

 


Gene deFuser

| Home | Retrieve Results | References | Help |
| --- | --- | --- | --- |

Back to Main Results of Job Final\_Tet

# Query Name: 3692.m00067

Candidate fusion gene

## Query Sequence:

MSSKEPSKKENLWNNILNETGQKNKFQNKQVLVLGTRGTGKRTLIDSLHEISQTIYPKIRSNEMQSGKLRIAGSVSALDYVYLNVKNLDDQDEDTMAKLGFYIIDEETQSQLLPIVLKQNTVKDTLITIMLDLSEPWTFMEQLDKWIEMITNHLQSLQINLETLDQMRAKVEKLFRDYKEPVLDSDGKLVKSDTQISPEEEAELIKLKSSVPLPEGSLTSNLVYKETYRFLNQIKSDLIAQSNDQQSQIVLYYLRKYCLQYGASLVFTSSKNQESLEIYYEYMLHTLYGFKNKNKSEIYTIDSIFIPSGSDNLNLLKQSNSTIFEQCDSKPYEELVKRPTSKNQRQEEVVCEDEQEFLKNLVNDKSNPEPIQSAKSSISAILQSGTSSTSTRTVNPPIVGNQGTTSVAKFYQTLLNQPNDARRLRENAERDLQQRVGNSTNNYFRNDEIQQKQNYPIKYLNKHTVQQLIKCTKIKGVGKSCMLIRYSKNDFVTDYNATIGVEFQSKIVKINDASVKLQIWDTAGQESFRSIVKSFYKKTVGVLLVYNIANKESFQSIKHWLTEAKENSEESAIFVLVGAQSDREKDFCIKSRKISREEAEQFQKANQIDLFFEVSSKNNSFIDECFEKTAYAAFMKQVNQQLKKSFQGSSFISNNGNIRSHNQSVPIKEQSRRSSNKSGCC

### Significant Ortholog Group Hits and their Scores:

| N terminus | | C terminus | |
| --- | --- | --- | --- |
| [N] KOG3905 Dynein light intermediate chain | 26.6666666666667 | [U] KOG0081 GTPase Rab27, small G protein superfamily | 40 |
|  |  | [U] KOG0095 GTPase Rab30, small G protein superfamily | 40 |
|  |  | [R] KOG0088 GTPase Rab21, small G protein superfamily | 40 |
|  |  | [R] KOG0083 GTPase Rab26/Rab37, small G protein superfamily | 40 |
|  |  | [U] KOG0086 GTPase Rab4, small G protein superfamily | 40 |
|  |  | [R] KOG0080 GTPase Rab18, small G protein superfamily | 40 |
|  |  | [U] KOG0097 GTPase Rab14, small G protein superfamily | 40 |
|  |  | [TU] KOG0084 GTPase Rab1/YPT1, small G protein superfamily, and related GTP-binding proteins | 37.5486024484548 |
|  |  | [U] KOG0094 GTPase Rab6/YPT6/Ryh1, small G protein superfamily | 37.4133868724263 |
|  |  | [R] KOG0394 Ras-related GTPase | 37.1257869770987 |
|  |  | [U] KOG0093 GTPase Rab3, small G protein superfamily | 35 |
|  |  | [U] KOG0087 GTPase Rab11/YPT3, small G protein superfamily | 34.3324443860662 |
|  |  | [U] KOG0098 GTPase Rab2, small G protein superfamily | 31.0469542162855 |
|  |  | [R] KOG0091 GTPase Rab39, small G protein superfamily | 30 |
|  |  | [R] KOG0079 GTP-binding protein H-ray, small G protein superfamily | 30 |
|  |  | [TU] KOG0078 GTP-binding protein SEC4, small G protein superfamily, and related Ras family GTP-binding proteins | 28.6236164337682 |
|  |  | [U] KOG0092 GTPase Rab5/YPT51 and related small G protein superfamily GTPases | 28.4834922711294 |
|  |  | [T] KOG4423 GTP-binding protein-like, RAS superfamily | 27.4889883746241 |
|  |  | [T] KOG4252 GTP-binding protein | 24.8465253307803 |

#### Graphs (click to enlarge):

|  |  |
| --- | --- |
| BLAST of Query Sequence | Location of Ortholog Group Hits |
|  |  |

Contact: Andre Cavalcanti\_\_\_\_\_Last Modified September 14, 2010
